# West Nile Virus Experimental Evolution *in vivo* and the Trade-off Hypothesis

**DOI:** 10.1371/journal.ppat.1002335

**Published:** 2011-11-10

**Authors:** Eleanor R. Deardorff, Kelly A. Fitzpatrick, Greta V. S. Jerzak, Pei-Yong Shi, Laura D. Kramer, Gregory D. Ebel

**Affiliations:** 1 Department of Pathology, University of New Mexico, Albuquerque, New Mexico, United States of America; 2 Wadsworth Center, New York State Department of Health, Slingerlands, New York, United States of America; University of Texas at Austin, United States of America

## Abstract

In nature, arthropod-borne viruses (arboviruses) perpetuate through alternating replication in vertebrate and invertebrate hosts. The trade-off hypothesis proposes that these viruses maintain adequate replicative fitness in two disparate hosts in exchange for superior fitness in one host. Releasing the virus from the constraints of a two-host cycle should thus facilitate adaptation to a single host. This theory has been addressed in a variety of systems, but remains poorly understood. We sought to determine the fitness implications of alternating host replication for West Nile virus (WNV) using an *in vivo* model system. Previously, WNV was serially or alternately passed 20 times *in vivo* in chicks or mosquitoes and resulting viruses were characterized genetically. In this study, these test viruses were competed *in vivo* in fitness assays against an unpassed marked reference virus. Fitness was assayed in chicks and in two important WNV vectors, *Culex pipiens* and *Culex quinquefasciatus*. Chick-specialized virus displayed clear fitness gains in chicks and in *Cx. pipiens* but not in *Cx. quinquefasciatus. Cx. pipiens*-specialized virus experienced reduced fitness in chicks and little change in either mosquito species. These data suggest that when fitness is measured in birds the trade-off hypothesis is supported; but in mosquitoes it is not. Overall, these results suggest that WNV evolution is driven by alternate cycles of genetic expansion in mosquitoes, where purifying selection is weak and genetic diversity generated, and restriction in birds, where purifying selection is strong.

## Author Summary

Arthropod-borne viruses, including West Nile virus (WNV) have long been considered to be subject to a fitness “trade-off” because they must replicate in at least two taxonomically divergent hosts in order to perpetuate. Results of studies testing this trade-off hypothesis have been inconsistent and largely dependent on which virus family is studied and which experimental system used. Therefore, considerable ambiguity exists in the literature regarding how host alternation influences virus population biology. Accordingly, we allowed WNV to adapt to each of its main hosts (mosquitoes and birds) for 20 sequential passes in order to determine whether host alternation in nature imparts a fitness compromise, as predicted by the trade-off hypothesis. After host-specialization, passed viruses were competed *in vivo* in mosquitoes and chicks against an unpassed marked reference virus to determine whether replicative fitness gains or losses had occurred. Our results demonstrate that the trade-off hypothesis accurately predicts WNV adaptation in the avian environment but not in mosquitoes. Overall, our results suggest that WNV adaptation is controlled by alternating cycles of genetic expansion in a permissive mosquito environment and restriction in avians, where purifying selection is dominant.

## Introduction

West Nile virus (WNV, family *Flaviviridae: Flavivirus*) is an arthropod-borne virus (arbovirus) that has demonstrated remarkable success since being introduced to North America in 1999. Within three years after its introduction the virus had adapted to local mosquito vectors and within 8 years had become fully endemic [1], [2], [3]. Viruses with RNA genomes, like WNV, have higher mutation rates than those of most DNA viruses due to error-prone replication [4]. However, arboviruses seem to evolve more slowly compared to single-host RNA viruses [5]. The trade-off hypothesis is a commonly postulated theory suggesting that this slower rate derives from the biological requirement for alternating replication in two taxonomically divergent hosts (vertebrates and arthropods). Under the trade-off hypothesis, virus fitness in both hosts is reduced in comparison to single host viruses, which can “specialize” on a single host environment [Recently reviewed by Ciota and Kramer [6]]. Several studies have reported that releasing arboviruses from host alternation and allowing sustained replication in a single host results in rapid adaptation to the specialized host, often with a corresponding fitness loss in the bypassed host, providing support for the trade-off hypothesis [7], [8], [9], [10].

Nonetheless, considerable ambiguity exists in the literature concerning the impact of host alternation on arbovirus adaptation and fitness. Importantly, neither the receptor-ligand interactions most important for virus entry and tropism nor the intracellular resources that might form the basis for host specialization in a putative fitness “trade-off” are well understood. Most studies of the trade-off hypothesis have involved either flaviviruses or alphaviruses (family *Togaviridae*), two of largest, most medically relevant families of arboviruses. Results of these studies are inconsistent and seem to differ between virus families (alphaviruses vs. flaviviruses) and experimental systems (cell-culture vs. animals). Among flaviviruses, the trade-off hypothesis is often only partially supported. Host specialization frequently results in fitness increases; however, that these increases carry a fitness cost in the bypassed host is less well supported. Work in cell-culture with dengue virus (genus *flavivirus)* has shown that single-host-specialized virus replicated faster and reached higher titers in the specialized cell-line but reciprocal fitness losses were less extreme and inconsistent [10], [11]. Another cell-culture study found that mosquito cell-specialized WNV and St. Louis encephalitis virus (SLEV, *flavivirus*) displayed improved fitness and more rapid replication in mosquito cells with only modest and inconsistent fitness losses in chicken cells [12]. *In vivo* studies with flaviviruses have also been difficult to reconcile with the trade-off hypothesis. For example, chick-specialized SLEV showed increased infectivity in chicks but was unchanged in mosquitoes, while mosquito-specialized virus was unchanged in both systems [13]. Conversely, serial passage of WNV in mosquitoes resulted in faster replication and higher peak titers in mosquitoes with no significant cost to replication in live chicks [14]. The impact of extensive *in vivo* serial passage on fitness of WNV within biologically relevant hosts has been difficult to resolve because the determinants of virus fitness in either host (mosquitoes or birds) have been poorly understood. Recent advances have provided a more complete mechanistic understanding of *in vivo* fitness determinants that may shed light on phenomena previously attributed to “trade-offs”. For example, genetic diversification of WNV is driven by, and can circumvent, mosquito immune mechanisms [15]. Additionally, the avian environment applies purifying selection to virus populations, but mosquitoes do not [16], [17]. Observed fitness “trade-offs” may thus be partially attributable to diversity-permissive and -restrictive environments in mosquitoes and birds, respectively.

**Figure 1:**
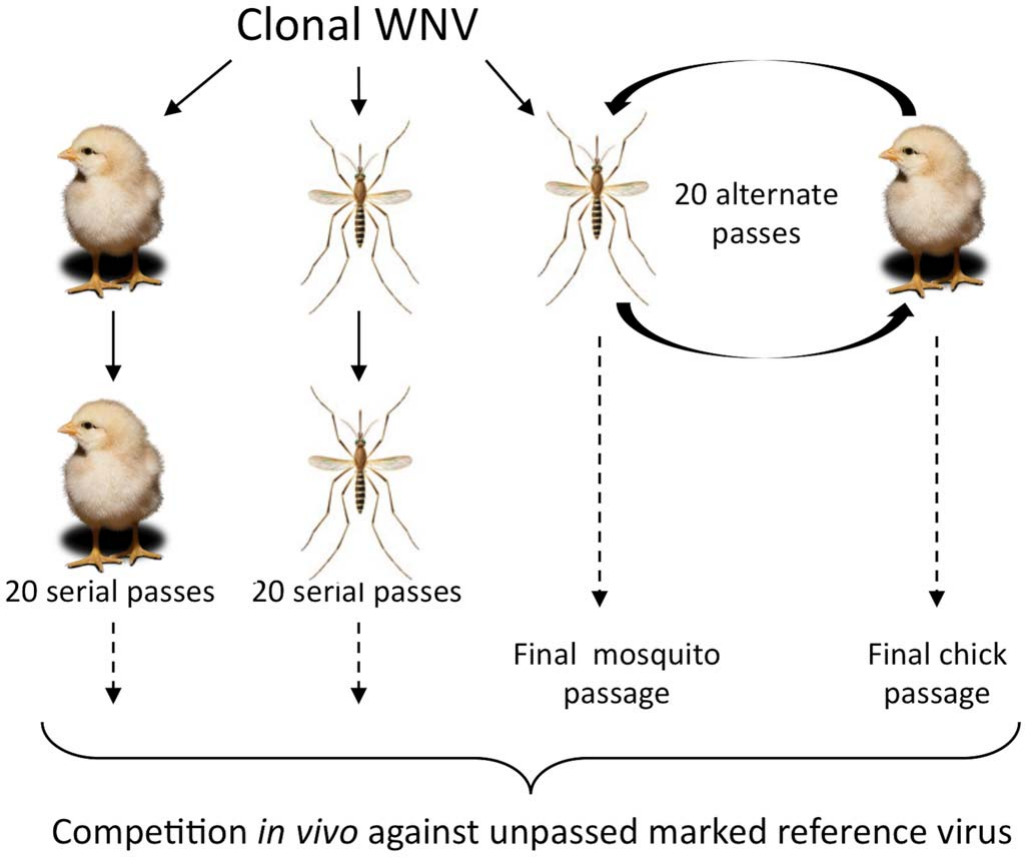
Serial and alternate passage experimental design. Virus derived from a WNV infectious clone was passed 20 times through chicks, 20 times through *Cx. pipiens* mosquitoes or 20 times alternating between the two (10 cycles). Each passage series was performed in triplicate and the final virus stocks were then used in *in vivo* competition assays to assess gains or losses in replicative fitness.

In light of this, we re-examined the trade-off hypothesis by determining the impact of host specialization on WNV fitness, here defined as the capacity for successful genome replication. In particular, (a) bird-specialized WNV, (b) mosquito-specialized WNV, (c) alternately passed WNV and (d) unpassed WNV, were competed against genetically marked WNV *in vivo* in mosquitoes and chickens. In previous studies we passed WNV exclusively in chicks or mosquitoes 20 times, or passed the virus alternately between mosquitoes and chicks a total of 20 times (figure 1) [17], [18]. In this study, the resulting WNV was competed against unpassed marked reference virus (WNV-REF), derived from the same clone used for passage initiation in order to determine whether host specialization leads to fitness gains and/or losses in the WNV system. Our studies, through the use of *in vivo* model animals for both passage and fitness determination, as well as triplicate performance of each treatment and the use of a higher passage number than typically used, provide a more representative model of the effect of host specialization on WNV fitness and the trade-off hypothesis, than has been possible with cell-culture models.

## Results

### Fitness in chicks

**Figure 2:**
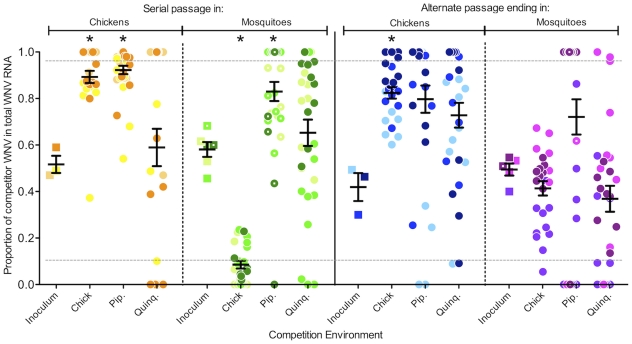
The trade-off hypothesis is not supported by results from *in vivo* competitions. After serial or alternate passage WNV strains were competed against a reference virus in chicks, *Cx. pipiens* mosquitoes (Pip.) and *Cx. quinquefasciatus* mosquitoes (Quinq.). Each of four treatments (serial passage in chicks, serial passage in mosquitoes, final chick passage of the alternate series, final mosquito passage of the alternate series) was performed in triplicate (represented by light, medium and dark shades of each color). Inocula (squares) contained approximately equal parts passed test virus and unpassed reference virus and were identical across cohorts except for 5 Pip. cohorts for which comparable inocula had to be re-created (points with white centers). Each cohort comprised 7–10 chicks or 9–11 mosquitoes with each animal represented by a circle. Mean proportions of test WNV for each cohort were compared with the inocula means in unpaired t-tests where P≤0.05 was considered significant (astrices). Bars indicate cohort mean and standard error of the mean. Dashed lines at 0.1 and 0.9 indicate the range of high accuracy for the quantitative sequencing assay used as determined by Fitzpatrick et al [21].

When competitions were conducted in chickens, serially and alternately passed WNV demonstrated clear fitness changes. Serial passage in chicks resulted in fitness increases compared to unpassed virus in the homologous host (table 1, figure 2, unpaired t-test, P = 0.0102). Conversely, after serial passage in mosquitoes WNV displayed significantly decreased replicative fitness in chicks (P = 0.0056). WNV from the alternating passage series also experienced significant fitness changes. Alternating passage that concluded in chickens demonstrated fitness gains (P = 0.0061) but alternating passage that concluded in mosquitoes did not (P>0.05). Unpassed WNV-WT (wild-type, unmarked WNV derived from the same clone used to generate the marked reference virus) had fitness similar to WNV-REF (table 1). Sera and brain tissue from all chick cohorts were also collected on day 5 post-inoculation and showed no significant difference from day 2 sera (data not shown).

**Table 1:** Combined average proportions of total WNV RNA comprised of competitor RNA after competition against a marked reference virus in chicks or mosquitoes.

	WT unpassed		Serial Chick		Serial Mosquito		Alternate Chick		Alternate Mosquito	
	Input 1	Output 2	Input	Output	Input	Output	Input	Output	Input	Output
Mean (Chicks)	0.68	0.55	0.52	0.89	0.53	0.09	0.42	0.82	0.49	0.41
SEM	0.00	0.01	0.04	0.03	0.04	0.02	0.06	0.02	0.05	0.03
n	1	8	3	25	3	25	3	24	3	26
p-value^3^	na		**0.0102**		**0.0056**		**0.0061**		0.3707	
Mean (*Cx. pip.*)	0.68	0.84	0.52	0.92	0.63	0.83	0.47	0.80	0.51	0.72
SEM	0.00	0.06	0.04	0.02	0.03	0.04	0.10	0.06	0.02	0.08
n	1	10	3	33	3	30	3	29	3	30
p-value	na		**0.0056**		**0.0291**		0.0586		0.3998	
Mean (*Cx. quinq*.)	0.72	0.72	0.52	0.59	0.53	0.65	0.42	0.73	0.49	0.37
SEM	0.00	0.07	0.04	0.08	0.04	0.06	0.06	0.05	0.05	0.06
n	1	9	3	31	3	30	3	28	3	29
p-value	na		0.7242		0.3975		0.0560		0.2702	

1 = input is the inoculum.

2 = output is either day 2 chick serum or day 7 whole mosquito homogenate.

3 = p-value was determined in an unpaired t-test between input and output for each cohort (significance is defined as p≤0.05 and is noted in bold).

### Fitness in mosquitoes

When competitions were conducted in *Cx. pipiens*, fitness changes also were observed (figure 2). Chick-passed WNV displayed significant replicative fitness increases over the unpassed marked reference virus (P = 0.0056). Additionally, after 20 serial passes in mosquitoes replicative fitness increases were observed (P = 0.0291). Neither the viruses from the alternate passage series nor the control WNV-WT exhibited significant changes in replicative fitness (table 1). In contrast to the results from the competition experiments in chicks and *Cx. pipiens* there were no significant replicative fitness changes for any of the serially or alternately passed viruses when competed against the unpassed marked reference virus in *Cx. quinquefasciatus* mosquitoes (table 1, figure 2).

### Relationship between genetic diversity and fitness

**Figure 3:**
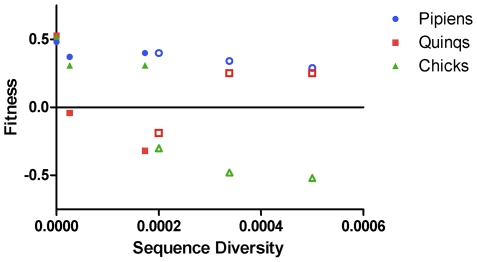
Intrahost genetic diversity is associated with decreased fitness in chickens but not mosquitoes. Fitness was computed as the difference between the test:REF ratio at input and after competition, such that numbers greater than zero indicate fitness increases and numbers less than zero indicate fitness declines. Sequence diversity was computed as the proportion of nucleotides in the test virus population with mutation, as described by Jerzak et al [18]. Fitness was measured in *Cx. pipiens* (blue circles) *Cx. quinquefasciatus* (red squares) and chickens (green triangles). Open symbols indicate passage history in mosquitoes, closed symbols indicate passage history in chickens. Sequence diversity was significantly negatively correlated with fitness in chickens (Spearman r = −0.9856, P = 0.0028).

To determine whether intrahost WNV genetic diversity was associated with fitness in the three hosts in which measurements were conducted, fitness was plotted as a function of viral genetic diversity (figure 3) and dN/dS (data not shown). Fitness was computed as the difference in proportion of test virus after competition compared to its proportion at input. Sequence diversity and dN/dS were computed as described previously [17], [18]. In mosquitoes, intrahost genetic diversity was not correlated with fitness (for *Cx. pipiens*, Spearman r = −0.8117, P>0.05, for *Cx. quinquefasciatus*, Spearman r = −0.05798, P>0.05). In chickens, diversity was significantly negatively associated with fitness (r = −0.9856, P = 0.0028). Similar results were obtained when the relationship between dN/dS and fitness was analyzed, with increasing dN/dS associated with lower fitness in chickens, but not mosquitoes (not shown).

## Discussion

Because arboviruses replicate in both arthropod and vertebrate hosts and seem to evolve more slowly than single-host RNA viruses, it is often proposed that they “trade” optimal fitness in either host in exchange for adequate fitness in both. Tests of this “trade-off” hypothesis most often consist of releasing a virus from host alternation and allowing it to specialize on one host or the other, then comparing fitness or genetic sequence data to the unpassed or alternately passed virus [9], [10], [13], [18], [19]. Due to the complexity of arbovirus transmission cycles, and in many cases the lack of appropriate *in vivo* models (for dengue virus, for example) these studies have largely been conducted *in vitro* in tissue culture, with inconsistent results. This lack of consistency appears to be related to differences in virus families, host species, passage regimes and approaches to measuring virus fitness. In most cases, the mechanistic basis for observed trade-offs have not been identified. Moreover, the diversity of experimental systems has made it difficult to identify the merits and defects of the trade-off hypothesis. Here, we used a completely *in vivo* approach to test whether or not WNV host alternation supports the trade-off hypothesis. By conducting the passage series and the competitions in relevant hosts *in vivo*, we sought to circumvent several of the caveats required in interpreting many previous studies. In sum, our data support the growing body of evidence that the trade-off hypothesis does not accurately predict WNV population dynamics [14], [20]. Interestingly, our findings are somewhat at odds with a similar literature developing in the field of alphavirus-host interactions, which tend to support the trade-off hypothesis [7], [8], [9], [19].The reasons for this are not entirely clear, but may be related to differences in virus replication *in vivo* (i.e. differences in host factors required for replication or host-cell receptor utilization). Differences in replication and/or mutation rates could also impact genetic diversity or population composition resulting in fitness changes. Additional comparative studies are required to develop a complete understanding of the underlying differences between fitness trade-offs in flaviviruses compared to alphaviruses.

Serial passage in chickens resulted in fitness gains in both chickens and *Cx. pipiens* mosquitoes, but fitness in a related mosquito species (*Cx. quinquefasciatus*) was unchanged. These results are at odds with the trade-off hypothesis because although the observed fitness increases in chickens would have been predicted, expected losses in the bypassed host (mosquitoes) were not observed. Notably, the WNV strains that had undergone sequential passage exclusively in chickens had patterns of nucleotide substitution suggesting that they were subject to strong purifying selection during replication in chickens [17]. In addition, intrahost genetic diversity in general was very low after passage in chickens. Collectively these observations suggest that during WNV replication in chickens, high overall fitness is maintained because deleterious mutations are rapidly removed by selection.

Serial passage of WNV in mosquitoes resulted in slight fitness gains in one species of mosquito (*Cx. pipiens*, the host in which the virus was sequentially passed), no change in a related species (*Cx. quinquefasciatus*), and extreme fitness losses in chickens. These findings seem to support the trade-off hypothesis. Purifying selection is relaxed in mosquitoes leading to high genetic diversity [16], [17]. It therefore seems likely that much of the genetic diversity generated during mosquito infection consists of mutations that are selectively neutral in mosquitoes but are slightly or strongly deleterious in the chick environment, leading to chick-specific fitness declines. This observation is supported by our analysis of the relationship between virus fitness and the genetic diversity within the WNV test population (figure 3). Moreover, our results suggest that the mechanistic basis for the observed fitness trade-off following mosquito passage is likely related to intrahost genetic diversity and different selective environments in each host type.

Alternating passage of WNV generally produced negligible fitness changes, largely in accordance with the trade-off hypothesis, although minor non-significant changes are apparent upon visual inspection of the data in figure 2. Possible explanations for adaptation in the absence of genetic coding change may include post-transcriptional modification or codon usage differences between the two environments (avian and mosquito) that may influence replication efficiency in the subsequent heterologous host. Concluding alternating passage in both mosquitoes and chicks also permitted us to examine the impact of two serial passes in each type of host. Interestingly, fitness gains were observed in chicks when they were the final host for alternating passage. These cohorts essentially represent two serial passes in chicks – the final pass of this series was in chicks and the subsequent competition was in chicks. After 20 alternate passes, two serial passes produced fitness gains comparable in magnitude to those observed after 20 serial passages. This finding underscores our understanding that purifying selection is extremely strong in these hosts.

Importantly, our passage regimen of 20 passes may not be robust enough to allow establishment of equilibrium for the virus populations being examined. Passage was terminated after 20 rounds because the logistics of serial passage become more restrictive when working with an *in vivo* model compared to an *in vitro* model. It is possible that with additional passage, the predictions of the trade-off hypothesis may be better satisfied in the invertebrate host. However, most data thus far indicates that mosquitoes are a diversity-permissive environment for WNV [17], [21]. Therefore, it is not clear how many additional passages would be required to achieve equilibrium in these hosts.

The trade-off hypothesis is only partially supported, and in a host-dependent manner, by our findings. When competed in chickens both single-host-specialized virus cohorts conformed to the predictions of the trade-off hypothesis; chick-specialized virus showed increased fitness and *Cx. pipiens* -specialized virus showed decreased fitness. Most of the data from this study, however, do not conform to the predictions of the trade-off hypothesis. When competed in mosquitoes, all chick-specialized as well as *Cx. pipiens*-specialized viruses displayed significant fitness gains. Whereas all passage series resulted in at least moderately improved fitness in *Cx. pipiens*, no passage series resulted in significant fitness changes in the *Cx. quinquefasciatus* environment. A similar study looking only at mosquito-specialized WNV also reports replicative fitness increases in mosquitoes without a corresponding cost in chicks [14].

Overall, these data suggest that the trade-off hypothesis, as conventionally stated, does not accurately predict WNV transmission dynamics because it fails to incorporate the mechanistic basis underlying fitness differences. Specifically, high mutational diversity of WNV increases fitness in mosquitoes by facilitating escape from their dominant RNAi-based antivirus response [15]. This fitness advantage in mosquitoes carries a selective cost in chickens because putative mosquito RNAi-escape mutations likely negatively impact virus replication.

Higher fitness gains for all four passage regimes were observed in *Cx. pipiens* compared to *Cx. quinquefasciatus*. These sibling species are primary vectors of WNV in the northern and southern United States, respectively [22]. Despite their close taxonomic relationship, differences have been noted previously in vector competence between the two species following feeding on WNV [1], [23], [24]. Mosquito passage of WNV was conducted in *Cx. pipiens*, which may account for the larger fitness increases observed during competitions in *Cx. pipiens*. However, this does not explain the puzzling extreme fitness gains for chick-specialized virus when competed in *Cx. pipiens*. We think it likely that after undergoing continuous purifying selection in the bird environment the virus replication is very efficient when it is then placed in the relatively permissive *Cx. pipiens* environment. It is possible that RNAi responses of differing magnitudes may contribute to the disparity observed for chick-specialized virus in the two mosquito species examined. It has been shown that the RNAi pathway in *Cx. quinquefasciatus* promotes genetic diversification [15], however no data for *Cx. pipiens* are available and the relative magnitudes of the RNAi have not yet been examined. Overall, our divergent results in *Cx. pipiens* and *Cx. quinquefasciatus* suggest that fitness determinants may be mosquito species-dependent.

In our studies, mosquito infection for passage and competition was achieved through intrathoracic (IT) -inoculation, which bypasses the mosquito midgut. This method was chosen because achieving adequately high virus titers for oral blood-feeding would require further passage of the virus in cell-culture and would potentially confound any effects of serial passage. Midgut infection and/or escape is considered a major bottleneck to vector infection by arboviruses [25].This restriction is likely specific to infection in general and not one that necessarily affects the genetic composition of the virus population achieving infection. Recently we have shown that genetic bottlenecks within *Cx. quinquefasciatus* do not significantly reduce WNV population diversity during horizontal transmission [26]. However, midgut infection and escape barriers cannot be entirely ruled out as influencing virus population genetics through, for example, selective constraints (i.e. as opposed to stocahstic effects). Because our infections were done by IT-inoculation, any selective constraints such as those imposed by transmission “barriers” in the natural transmission cycle were overcome. Nonetheless, the methods and approaches used to accomplish the passages described here allowed us to examine the impact of replication in divergent hosts in the absence of several factors (such as barriers and co-infections, for example) that would likely be present in nature.

The complete genome sequences for the endpoint viruses from all serially or alternately passed WNV lineages used in the current study have been previously published [17], [18]. Numerous synonymous and non-synonymous mutations were found in both structural and non-structural coding regions, but no signature mutations were found to be associated with any passage series and there was no data to suggest that adaptation had occurred. Importantly, individual virus isolates are known to comprise a mutant swarm that may contain minority genotypes not detectable in the consensus sequence that may exhibit dominant phenotypes [27]. Recent developments in deep sequencing technology will greatly facilitate future efforts at understanding the contributions of individual quasispecies to the overall fitness of arboviruses [28].

In conclusion, when released from the obligate cycling between avian and mosquito hosts, WNV experienced symmetrical fitness gains in specialized hosts but fitness losses in bypassed hosts were asymmetrical. In the avian environment fitness trade-offs are apparent and robust; however, in the mosquito environment no obvious fitness trade-offs were observed. These data are consistent with previously published work showing that the mosquito environment permits a much higher level of viral genetic diversity than the avian environment [16], [17]. Our results add to a growing amount of evidence that arboviruses in general do not fall into an intuitive pattern represented by the host trade-off assumption. WNV adaptation and evolution therefore seem likely to be driven by alternating between diversity-permissive and diversity-restrictive environments in the invertebrate and vertebrate hosts. Mosquito infection enables the development of genetic diversity and novel variants of WNV, while infection of birds applies purifying selection that maintains high replicative fitness.

## Materials and Methods

### Ethics statement

Experiments involving animals were conducted in accordance with protocols approved by the University of New Mexico Institutional Animal Care and Use Committee in strict adherence to recommendations set forth in the Guide for the Care and Use of Laboratory Animals of the National Institutes of Health (Assurance No. A3350-01), and approved by the University of New Mexico IACUC (protocol # 100450).

### Viruses and passage series

The wild-type virus (WNV-WT) that was used to initiate all passage series was derived from a WNV infectious clone as previously described [29]. The genetically marked reference virus (WNV-REF) was also derived from a WNV infectious clone and has been previously characterized [21]. This reference virus is identical to WNV-WT except for five sequential non-coding changes in the NS5 region of the genome from nucleotide positions 8313–8317. These changes were engineered using site-directed mutagenesis as described previously [30] and changed the parental sequence CTC TCA CGG to CTa agc aGG without altering the amino acid sequence or the replication kinetics and infectivity of the virus [21]. Viral RNA for WNV-WT and WNV-REF was electroporated into baby hamster kidney (BHK) cells and progeny virus was harvested directly and used without further cell-culture passage.

Serial and alternate passage of WNV-WT in chicks and mosquitoes has been previously described [17], [18] (figure 1). Briefly, 1–3 day old chicks [Charles River Specific Pathogen Free Avian Services (Franklin, CT) or Sunrise Farms (Catskill, NY)] were inoculated with 100 times the ID50 in three replicate concurrent lineages. At day 2 post-inoculation, serum was harvested, titrated to determine the correct dilution for re-inoculation and used to inoculate 100 times the ID50 into the next round of 1–3 day old chicks. After 20 such serial passes, the end-point sera for each of the three replicate concurrent lineages were harvested, titered and stored at −80°C until competition experiments were conducted. Adult, female *Cx. pipiens* mosquitoes (colony derived from larvae collected in Pennsylvania and maintained at the Wadsworth Center Arbovirus Laboratories since 2002) were IT-inoculated with 100 times the ID50. Three concurrent replicate lineages were maintained, with approximately ten individual mosquitoes per replicate inoculated. At day 7 post-inoculation, individual mosquitoes were triturated and homogenates were clarified by centrifugation, titrated to determine the correct dilution for re-inoculation, and used to inoculate 100 times the ID50 into the next cohort of mosquitoes. A single mosquito with the median viral load was selected for further passage. After 20 such serial passes, the end-point homogenates were titrated and stored at −80°C until competition experiments were conducted.

Alternating passage was also conducted in three concurrent replicate lineages and was begun with *Cx. pipiens* IT-inoculation as described above. After trituration, clarified homogenates were used to inoculate 100 times the ID50 into chicks as described above. Day 2 chick serum was then used to inoculate 100 times the ID50 into the next cohort of *Cx. pipiens* and alternate passage continued for 10 complete cycles or 20 total virus passes. Alternating passage was concluded in each host type to evaluate the possibility that a single round of replication in one or the other host might influence virus fitness.

After passage, the resulting viruses were characterized with respect to both complete genome sequence and population diversity. Results of these studies are described in detail in previous publications [17], [18]. Briefly, complete genome sequences were unremarkable, with no consistent changes noted at the genome level. However, purifying selection was associated with exclusive passage in chickens or alternating passage, with mosquito-passed WNV lacking evidence of purifying selection. Intrahost genetic diversity was related to passage history, with higher diversity associated with exclusive passage in mosquitoes, or alternating passage, but not with exclusive passage in chickens. These genetically characterized, passed viruses constitute the “test” viruses for competition studies. The inocula for competitions were created by mixing equal number of plaque forming units (pfu) of WNV-REF and passed “test” WNV. In most cases the inocula were prepared in advance, aliquoted and stored at −80°C with a fresh aliquot used for each competition. In 5 of the 13 *Cx. pipiens* competitions the inocula were re-created and are comparable to those used in the corresponding chick and *Cx. quinquefasciatus* competitions.

### Fitness competitions in chicks

Chicks were reared and competitions were performed in the University of New Mexico's animal biosafety level-3 (ABSL-3) laboratory. Specific-pathogen-free eggs were incubated and chickens hatched and maintained as described above and elsewhere [17], [18], [21]. At approximately 24 h post hatching, chicks were subcutaneously inoculated in the cervical region with 100 µL (2.5×10^2^–2.5×10^5^ total pfu) of mixed 1∶1 test∶REF WNV in animal inoculation medium (endotoxin- and cation-free phosphate buffered saline with 1% FBS) then returned to their brooders. At 48 h post-inoculation approximately 50 µL blood was collected in heparinized capillary tubes after brachial venipuncture. Serum was separated and used for viral RNA isolation. Experiments involving animals were conducted in accordance with protocols approved by the University of New Mexico Institutional Animal Care and Use Committee in strict adherence to recommendations set for in the Guide for the Care and Use of laboratory Animals of the National Institutes of Health (Assurance No. A3350-01), and approved by the University of New Mexico IACUC (protocol # 100450).

### Competitions in mosquitoes

Mosquitoes were reared and competitions were performed in the University of New Mexico's bio-safety level-3 (BSL-3) insectary. *Culex* mosquitoes were obtained from colonies at the University of New Mexico and the Wadsworth Center, New York State Department of Health. Mosquitoes were maintained at 27°C with a 16∶8 L∶D photoperiod and were used in competitions at 3–7 days post-emergence. Mosquitoes were anesthetized with CO_2_ and were IT-inoculated with 70–840 nl (2–18 total pfu) of mixed 1∶1 test∶REF WNV in animal inoculation medium using a Nanoject II (Drummond Scientific Company, Boomall PA). Inoculated mosquitoes were incubated in quart-sized cardboard containers with water and 10% sucrose provided *ad libitum*. At 7-days post inoculation whole individual mosquitoes were triturated using a TissueLyser (Qiagen Inc., Valencia, CA) and homogenates were clarified by centrifugation then used for viral RNA isolation.

### Genotype proportion determination

Total viral RNA was isolated from chick sera or mosquito homogenates using the RNeasy RNA Purification Kit (Qiagen Inc., Valencia, CA). Reverse-transcriptase polymerase chain reaction (RT-PCR) was then performed as previously described [21]. Briefly, one-step RT-PCR was performed using the SuperScriptIII system (Invitrogen Corporation, Carlsbad, CA) and primers designed to amplify an 853 base-pair region containing the 5-nucleotide genetic marker. Amplicon DNA was then purified using the QIAquick PCR Purification Kit (Qiagen Inc., Valencia, CA) and sequenced by the Sanger sequencing method (Genewiz Inc., South Plainfield, NJ). Sequence chromatograms were analyzed using the polySNP program (http://staging.nybg.org/polySNP.html) as described elsewhere [21], [31]. The proportion of each genotype was then computed for each of the 5 nucleotide positions and the mean proportion of all 5 is reported as the overall proportion of each genotype in the DNA sample. Comparison of relative proportions for input (inocula) and output (chick sera or mosquito homogenates) was done by an unpaired t-test performed in the GraphPad software package. A t-test P-value of ≤0.05 was interpreted as statistically significant. The measure of competitive fitness as determine by quantitative sequencing was limited by the range of detection which was previously determined to be 10–90% [21]. Thus the virus that “loses” in the competition may still be present in biologically significant proportions despite being undetectable by the quantitative sequencing assay.

### Virus assays

Before and after inoculation into chicks or mosquitoes, virus preparations were titered to determine the amount of total WNV (test-WNV plus WNV-REF). Inocula were serially diluted 10-fold in cell-culture medium [Eagle's minimum essential medium (MEM) containing 10% heat-inactivated fetal bovine serum (FBS), penicillin/streptomycin (100 units/mL), gentamicin (50 µg/mL), L-glutamine (1×) and fungizone (125 ng/mL)], then adsorbed onto confluent monolayers of Vero cells at room temperature for 60 min with continual rocking. Cells were then overlaid with cell-culture medium containing 0.4% agarose and maintained at 37°C with 5% CO_2_. After 48 h a second overlay of cell-culture medium containing 0.4% agarose and neutral red (66 µg/mL) was applied and incubated for an additional 24 h before plaques became apparent and were counted for virus titer calculation.
